# The effect of dietary intake of antioxidant micronutrients on burn wound healing: a study in a tertiary health institution in a developing country

**DOI:** 10.1186/s41038-015-0012-x

**Published:** 2015-08-12

**Authors:** Mary Adjepong, Pius Agbenorku, Patricia Brown, Ibok Oduro

**Affiliations:** 1 Department of Food Science and Technology, College of Science, Kwame Nkrumah University of Science & Technology, Kumasi, Ghana; 2 Reconstructive Plastic Surgery & Burns Unit, Department of Surgery, Komfo Anokye Teaching Hospital, School of Medical Sciences, College of Health Sciences, Kwame Nkrumah University of Science & Technology, Kumasi, Ghana

**Keywords:** Dietary intake, Antioxidants, Micronutrients, Recovery, Wound infection, Wound healing

## Abstract

**Background:**

Burn injury results in emotional stress affecting dietary intake and antioxidant micronutrient intake, which is known to have effects on recovery outcomes. The study aimed to assess dietary intake of antioxidant micronutrients and recovery outcomes of burn patients.

**Methods:**

Questionnaires were administered to 40 burn patients at Komfo Anokye Teaching Hospital (Ghana) from January 1, 2014 to May 30, 2014. The data taken include anthropometric measurements and dietary assessment. Their nutrient intakes were assessed with the Nutrient Analysis Template. The average intakes were compared to the recommended daily allowance. Assessment of recovery was based on records of wound healing assessments and infection rates from the health practitioners.

**Results:**

A cross-sectional study of 40 patients revealed an average total burn surface area (TBSA) of 31.4 %, where 70.0, 35.0, 75.0, 52.5, 12.5 and 32.5 % patients were deficient in vitamins A, C and E, zinc, copper and selenium, respectively and adequate amounts of vitamin C intake were related with significantly better wound healing progress. Positive wound healing outcomes were observed for patients with adequate vitamins A and E and zinc intake. Less infection presented among patients with adequate amount of vitamins A and C and zinc, but this was not observed for patients with adequate vitamin E, copper and selenium.

**Conclusions:**

Most burn patients did not meet their dietary requirements for antioxidant micronutrients and this was due to meals not tailored to suit individual requirements. Adequacy of the antioxidants especially vitamin C resulted in positive wound healing outcomes. Hence, there is need for planned well-balanced meals of high vitamin C.

## Background

Burns place a great socio-economic burden on individuals, their families and the health services [1]. Apart from the financial burden to families, communities, and the nation, it leads to pain, infection, extensive scarring, wound and scar contractures, amputations and death in some of its victims [2].

The World Health Organization (WHO) estimates that 43,000 people die of burns in Africa every year with a rate of 6.1 per 100,000 [2]. In Ghana, the incidence is 6.1 per 100,000 persons, an extrapolated incidence of 1.3 % with more women and children being victims because of the roles of a typical Ghanaian woman. Burn injury accounts for 265,000 deaths annually and children below 20 are the most affected. It is important to note that majority of deaths occur in low- and middle-income countries [3].

The main causes of mortality in burn patients are associated with infection, delayed wound healing, and extensive protein catabolism. Underlying these processes include immunological, endocrine, inflammatory and other metabolic responses [4]. When the skin is exposed in burn patients, there are several mechanisms that lead to oxidative damage to the cell membranes. This includes the peroxidation of polyunsaturated fatty acid residues in membranes that can lead to membrane impairment, in addition to fragmentation of proteins at vulnerable points in the amino acid chain. There is also the irreversible damage to protein sites where metal ions normally bind to functional protein [5].

Apart from this destruction to the cell membrane, there are several metabolic responses that render the individual susceptible to malnutrition. Among them is hypercatabolism, which contributes to the breakdown of muscle and fat. Hypermetabolic response in the individual leads to an increase in the basal metabolic rate to about 180 % and an increase in the utilization of nutrients [6] and can last at least 9 to 12 months [7]. The increase in the production of catecholamines contributes to an increase in the resting energy expenditure. Similarly, the release of glucocorticoids leads to an alteration in protein and carbohydrate metabolism. These result in an increase glucagon/insulin ratio leading to hyperglycemia. This is a result of increased gluconeogenesis, proteolysis, glycogenolysis and lipolysis [6]. There is also nutrient loss in urine and wound exudates and this can lead to hypoalbumenuria [8].

Being a central part of post burn management, medical nutrition therapy ensures adequate nutrients for the victim to improve wound healing, to restore cutaneous losses and to manage infections [9]. Apart from the role of macronutrients in these, the role of micronutrients cannot be ignored especially antioxidant micronutrients.

Copper, zinc, selenium, and vitamins A, C and E are antioxidant micronutrients that are beneficial to burn patients when intakes are adequate. This is due to their roles in scavenging free radicals in the wound healing process—homeostasis, inflammatory phase, proliferative phase and remodeling—as well as their role in fighting infections [10, 11].

The aim of this study therefore was to assess the effect of dietary intake of antioxidant micronutrients on the various recovery outcomes of burn patients. The micronutrients considered were copper, zinc, selenium and vitamins A, C and E. The recovery outcomes are the progress of wound healing, presence of infection and the length of stay in the hospital.

## Methods

### Study setting

The Komfo Anokye Teaching Hospital is the second largest hospital in Ghana. It is the main referral hospital for the northern sector of Ghana. With a bed capacity of over a thousand, there are numerous units in the hospital that provide health care for persons who come to the facility. Among the units are the Reconstructive Plastic Surgery and Burns Unit under the Directorate of Surgery. In this Unit is a Burns Intensive Care Unit (BICU) that caters to burn patients who are critically ill. There are other wards that cater to less severe cases.

### Subjects

The chosen subjects were burn patients who were admitted to the BICU from January to May 2014 of all ages who consented to the study. Some other cases of interest were also taken from the outpatient department.

### Data collection

Pretested questionnaires were administered to clinically consenting patients. Demographics, medical history, anthropometry, food frequency, and triplicate dietary recalls were done. Anthropometric measurements include weight, height, and mid-upper arm circumference for children. Multiple dietary recalls were done for all patients for 3 days including a day in a weekend. On admission, patients were fed with meals provided by the hospital.

Using handy measures, the weights of all the foods consumed by the subjects were recorded. The nutrient compositions in the foods were analyzed using the Nutrient Analysis Template (University of Ghana, Food Science and Nutrition Department). The average of the intakes was recorded and compared to the recommended daily intakes (University of Ghana, Food Science and Nutrition Department) of the individuals. Assessment of recovery was based on records of wound healing assessments, infection rates and the length of stay in the hospital from the health practitioners and a tape measure was also used to measure the diameter of the wounds.

### Data analysis

Data storage was done using Microsoft Excel 5.0 and graphs were drawn using Microsoft Excel. Chi-square test was used to compare the categorical parameter/qualitative variable among the groups and *p* < 0.05 was considered statistically significant.

### Ethical clearance

Ethical clearance was granted by the Committee of Human Research Publication and Ethics of Komfo Anokye Teaching Hospital (KATH)/School of Medical Sciences, Kwame Nkrumah University of Science and Technology (KNUST), Kumasi.

## Results

### Demographic data

In Table 1, the demographic statistics of burn patients at KATH from January to May 2014 is shown. Out of the 40 patients, 23 of them were aged above 14 years representing the highest age range followed by those who were aged between 2 and 14 years with 9 subjects. The lowest reported age range was for patients below 2 years. The results also showed more males suffering from burn injury (65 %) than females (35 %). Only two subjects, representing a total of 5 %, had a level of tertiary education, with majority of the subjects having no education (30 %), primary level of education (37.5 %), and secondary education (27.5 %). In view of this, it was not surprising that 5 % of the subjects were professionals in their fields with the rest being artisans (25 %) and traders (22.5 %) and a majority being unemployed (47.9 %).

**Table 1:** Demographic profiles of 40 burn patients at KATH BICU

Characteristics	Number of patients	
	*N* (total = 40)	%
Age (years)		
<2	8	20.0
2–14	9	22.5
>14	23	57.5
Sex		
Male	26	65.0
Female	14	35.0
Level of education		
Primary school	15	37.5
Middle/junior high school	8	20.0
Senior high school	3	7.5
Tertiary 1 to 3 years (diploma/certificate/professional)	1	2.5
Tertiary 4 or more years (degree/postgraduate/professional)	1	2.5
None	12	30.0
Occupation		
Artisan	10	25.0
Professional	2	5.0
Trader	9	22.5
Unemployed	19	47.5

### Anthropometric and medical information of the patients

Majority of the patients (adults) in this study had normal basal metabolic rate, with fewer (7.5 %) of them being obese. The major cause of burn injury in this study was thermal causes: scalds, open flame, and explosions. Chemical and electric causes recorded minimum injury, 2.5 and 5.0 %, respectively. Half (50.0 %) of the patients suffered severe injury of above 25.0 % total burn surface area (TBSA), followed by 10–25 % TBSA with 27.5 % of the patients. In addition, majority of the patients (50 %) suffered partial thickness burns. The results are shown in Table 2.

**Table 2:** Anthropometric and medical information of patients

Characteristic	Number of patients	
	*N* = 40	%
Description of basal metabolic index (BMI)^a^		
Underweight	0	0
Normal	13	32.5
Overweight	7	17.5
Obese	3	7.5
Cause of burn		
Electrical	2	5.0
Chemical	1	2.5
Thermal	37	92.5
Total burn surface area (TBSA)		
<10 %	9	22.5
10–25 %	11	27.5
>25 %	20	50.0
Severity of burn		
Superficial thickness	12	30.0
Second degree/partial thickness	20	50.0
Third degree/full thickness	3	7.5
Mixed thickness	5	12.5

^a^Mid-upper arm circumference (MUAC) was the nutritional assessment tool for children (14 years and below); all the children were within normal ranges

### Adequacy and inadequacy of dietary antioxidant micronutrients

When dietary intake of antioxidant micronutrients was assessed and compared to the recommended dietary allowances of the population based on the ages, the following results were obtained: 30.0 % of the patients had adequate vitamin A and 70.0 % had inadequate intakes in their diet, 65 % had adequate intakes of vitamin C with 35.0 % having inadequate intakes and 25.0 % of the patients had adequate vitamin E intakes with 75.0 % recording inadequate intakes.

With regard to trace element intake in the diet, the following results were recorded: 47.5, 90.0 and 67.5 % had adequate dietary intake of zinc, copper, and selenium, respectively, with 52.5, 12.5, and 32.5 % of the patients recording inadequate dietary intake (Table 3).

**Table 3:** Dietary intake of antioxidant micronutrients and wound healing progress

	*N* (total = 40)	Week 1 wound healing			Week 2 wound healing		
		*n*	(%)	*p* value	*n*	(%)	*p* value
Vitamin A							
Adequate	12	10	(83)	0.162	7	(58)	0.629
Inadequate	28	17	(61)		14	(50)	
Vitamin C							
Adequate	26	21	(81)	0.015	16	(62)	0.119
Inadequate	14	6	(43)		5	(36)	
Vitamin E							
Adequate	9	7	(78)	0.455	5	(56)	0.835
Inadequate	31	20	(65)		16	(52)	
Zinc							
Adequate	19	15	(79)	0.141	11	(58)	0.516
Inadequate	21	12	(57)		10	(48)	
Copper							
Adequate	36	24	(67)	0.736	18	(50)	0.342
Inadequate	4	3	(75)		3	(75)	
Selenium							
Adequate	26	19	(73)	0.305	13	(50)	0.666
Inadequate	14	8	(57)		8	(57)	

*p* < 0.05 was considered statistically significant

### Dietary intake of vitamin A and wound healing progress

The measure of the recovery outcome considered was the length of hospital stay and the presence of infections. The length of stay in the hospital was dependent on the TBSA of the patient. The study of the wound healing outcomes for 2 weeks revealed that out of the 12 patients who had adequate dietary intake of vitamin A, 83.3 % of them had a positive wound healing outcome during the first week with 58.3 % of the patients recording positive wound healing outcomes in the second week. When this is compared to 28 patients with inadequate dietary intake of vitamin A, it can be observed that 60.7 and 50.0 % of the patients had positive wound healing outcomes, showing a decrease in the wound healing outcomes. When they were compared statistically, the *p* values showed that there was no statistical difference in the outcomes in weeks, 0.162 and 0.629 when patients with adequate intake and inadequate intake were compared.

### Dietary intake of vitamin C and wound healing progress

Similarly, the wound healing outcomes for patients with adequate intake of vitamin C showed a remarkable progress in wound healing. Out of 26 patients, 80.8 and 61.5 % of them recorded positive wound healing outcomes for weeks 1 and 2, respectively. In contrast, there was progress in wound healing for only 42.9 and 35.7 % of the patients with inadequate dietary intake of vitamin C for the first and second weeks, respectively. Also, there was statistical significance between vitamin C intake and wound healing in week 1 with a *p* value of 0.015 when patients with adequate intake and inadequate intake were compared.

### Dietary intake of vitamin E and wound healing progress

The role of vitamin E in the progress of wound healing is outstanding. It was observed that the 9 patients with adequate intake of vitamin E recorded positive outcomes of 77.8 and 55.6 % in wound healing in the first and second weeks, respectively, as compared to the 31 patients with inadequate dietary intake of the vitamin who recorded 64.5 and 51.6 % in their wound healing outcomes. When they were compared statistically, the *p* values showed that there was no statistical difference in the outcomes in weeks, 0.455 and 0.835 when patients with adequate intake and inadequate intake were compared.

### Dietary intake of zinc and wound healing progress

There was a remarkable wound healing progress in the patients who had adequate dietary intake of zinc. There was a corresponding increase in the number of patients with regard to their wound healing outcomes as seen in the previous antioxidants; 79.0 and 57.9 % of the patients recorded positive progress in wound health out of the 19 patients who had adequate dietary intake of the trace element as compared to those with inadequate dietary intake of the trace element who recorded 57.1 and 47.6 % in their wound healing outcomes. When they were compared statistically, the *p* values showed that there was no statistical difference in the outcomes in both weeks, 0.141 and 0.516 when patients with adequate intake and inadequate intake were compared.

### Dietary intake of copper and wound healing progress

The adequacy in the dietary intake of copper also yielded remarkable wound healing outcomes. The first and second weeks recorded a positive wound healing rate of 66.7 and 50.0 % for the 36 patients with adequate dietary intake of copper. Patients with inadequate amounts of this trace element in their diet recorded a relatively higher progress in the wound health outcomes recording 75.0 % for each week. When they were compared statistically, the *p* values showed that there was no statistical change in the outcomes in both weeks, 0.736 and 0.342 when patients with adequate intake and inadequate intake were compared.

### Dietary intake of selenium and wound healing progress

The adequacy of selenium in the diet of patients follows the same trend as the other antioxidant nutrients. There was a progress in the wound healing outcomes in the first and second weeks for patients who had adequate intake of selenium in their diet. It was also recorded that there was a relative decrease in the number of patients who had inadequate amounts of selenium in their diet. When they were compared statistically, the *p* values showed that there was no statistical change in the outcomes in both weeks, 0.305 and 0.666 when patients with adequate intake and inadequate intake were compared.

### Adequacy of antioxidant vitamins and infection

**Fig. 1:**
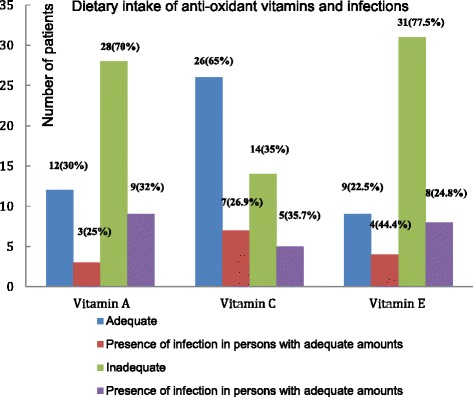
Intake of antioxidant vitamins and infection

The rate of wound infection in patients with adequate dietary intake of vitamin A was 25.0 % compared to patients who had inadequate dietary intake recording a relatively higher figure, 32.0 %. Patients with adequate dietary intake of vitamin C also recorded a lower rate (26.9 %) of infections compared with patients with inadequate dietary intake (35.7 %). Though there were contrasting results, patients with adequate vitamin E recorded a wound healing rate of 44.4 % compared with their counterparts with inadequate amounts recording a lower rate of infection (24.8 %) as shown in Fig. 1.

### Adequacy of antioxidant trace elements and infection

**Fig 2:**
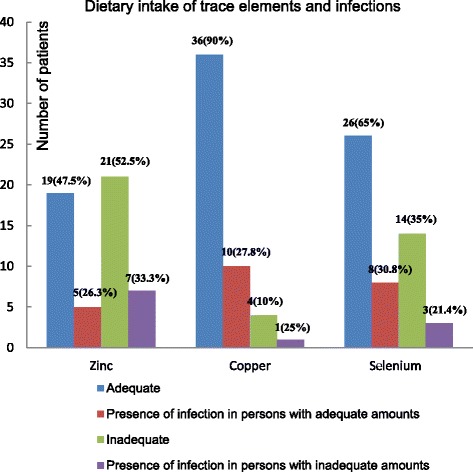
Dietary intake of trace elements and infections

Patients with adequate dietary intake of zinc recorded a lower rate of infection (26.3 %) as compared to patients with inadequate amounts. With regard to copper and selenium, the infection rate was relatively lower in patients with inadequate amounts (Fig. 2).

## Discussion

Children aged 14 and below are known to have a high incidence of burn injury in developing countries, and this could be a result of the roles played by most children with their mothers in the household [12]. Though children in this category did not record the highest figure; they recorded a considerably high percentage (42.5 %) of the total number of patients, giving the impression that the child within this category needs to be protected from burn injury. Also, children below age 2 years recorded 20.0 % for the burn injury which is quite high for such an age group. This could be as a result of negligence of mothers as well as the exposure of children to open flames in a typical setting of a home in a developing country [13]. Also, the socio-economic background has an effect on burn injury. Only 5.0 % of the participants of this study belonged to a high socio-economic class corresponding to works done by Bell et al. [14] which states that burn injury increases substantially with a lower socio-economic status.

The major cause of burn in this study was thermal (92.5 %) comprising of open flames, scalds, and gas explosion with chemical (2.5 %) and electrical (5.0 %) causes. This corresponds to the works done by Agbenorku et al. [15]. The results from this study also show that majority (50.0 %) of the patients suffered burns with a TBSA above 25.0 % which is quite alarming because most fatalities occur in such severities. In addition, 70.0 % of the participants had burn injury of second degree, third degree, and mixed thickness, giving an indication that most of the injuries were very severe. In their study, Gokdemir et al. [16] reported that mortality increases with increasing TBSA and burn depth. Also, the presence of ascobate in leukocytes and its rapid expenditure during infection and phagocytosis suggest that vitamin C has a role in immunity, hence the results obtained.

Vitamin C (ascorbic acid) influences all phases of wound healing such as the inflammatory phase, proliferative phase, and maturation, synthesis, and degradation of collagen [10]. Collagen synthesis, maturation and stabilization are also roles of vitamin C. It is also responsible for neutrophil migration to the site of injury and has a role in clotting when the skin is exposed. From this study, it was noted that during the first 2 weeks of monitoring the patients, patients with adequate dietary intake of vitamin C recorded a remarkable increase in wound healing. This corresponds to the work done by Barbosa et al. [17] and Sahib et al. [18] where supplementation of vitamin C reduced wound healing time. The results also revealed a significant change with a *p* value of 0.015 in the first week indicating that there was a significant improvement in wound healing outcomes in the first week.

Vitamin A is known to play diverse roles in the wound healing cascade. It enhances early inflammatory phase and promotes epithelial cell differentiation, collagen deposition, and immunity [10]; hence, patients with adequate dietary intake had progress in wound healing during the first 2 weeks. Also, intake of vitamin A results in an increase in lymphocyte proliferation, and this has the ability to prevent wound infection due to the reduction of microbes on wounds. Vitamin A is also known to aid in the repair of damaged tissue and hence may be beneficial in counteracting free radical damage [19], increasing the wound healing rate.

When a wound is exposed, the presence of microorganisms greater than 100,000 results in no epithelialization. Vitamin E is responsible for the preservation of macrophages which act by eliminating the microorganism by phagocytosis [10]. Due to its role in immunity, it is not surprising that patients with adequate amount in their diet had a good wound healing outcome as compared to patients with inadequate amounts in their diet. This is similar to works done by Barbosa et al. [17] and Sahib et al. [18].

The effect of adequate intake of trace element supplementation on the recovery of burn patients is noted in most studies. This is evidenced by reduced length of stay in hospitals, increase in protein turnover, as well as a reduction in infection. The body’s antioxidant defense network is premiered by glutathione peroxidase, and selenium is known to be a major component of glutathione transferase enzyme, an important enzyme for this antioxidant [19]. Though the roles of these are not fully known, their role on various recovery outcomes is prominent. Copper and zinc are also co-factors of superoxide dismutase enzyme [20]. The results obtained from this study revealed that patients who had adequate zinc, copper, and selenium in their diet had positive wound healing outcomes and patients with adequate zinc had fewer reports of infections. This corresponds with works by Berger et al. [4, 21].

## Conclusions

Most burn patients did not meet their dietary requirements for antioxidant micronutrient and this was due to meals not tailored to suit individual requirements, hence the need for planned and well-balanced meals. Routine supplementation of antioxidant micronutrients can also be considered. Policies to provide meals to burn patients on the ward are worth noting: vitamin C is the only micronutrient that showed a promising significant value (0.015) with respect to wound healing outcomes; hence, it is recommended that food with high vitamin C such as fruits and vegetables should be distributed to burn patients on admission.
